# Lipidome in colorectal cancer

**DOI:** 10.18632/oncotarget.7960

**Published:** 2016-03-07

**Authors:** Guifang Yan, Liqi Li, Bo Zhu, Yongsheng Li

**Affiliations:** ^1^ Institute of Cancer, Xinqiao Hospital, Third Military Medical University, Chongqing, China; ^2^ Department of General Surgery, Xinqiao Hospital, Third Military Medical University, Chongqing, China; ^3^ Center for Experimental Therapeutics and Reperfusion Injury, Department of Anesthesia, Perioperative and Pain Medicine, Brigham and Women's Hospital and Harvard Medical School, Boston, MA, USA

**Keywords:** lipidome, metabolism, colorectal cancer, fatty acid, cholesterol

## Abstract

Colorectal cancer (CRC) is the second leading cause of cancer-related deaths. Understanding its pathophysiology is essential for developing efficient strategies to treat this disease. Lipidome, the sum of total lipids, related enzymes, receptors and signaling pathways, plays crucial roles in multiple cellular processes, such as metabolism, energy storage, proliferation and apoptosis. Dysregulation of lipid metabolism and function contributes to the development of CRC, and can be used towards the evaluation of prognosis. The strategies targeting lipidome have been applied in clinical trails and showed promising results. Here we discuss recent advances in abnormal lipid metabolism in CRC, the mechanisms by which the lipidome regulates tumorigenesis and tumor progression, and suggest potential therapeutic targets for clinical trials.

## INTRODUCTION

Lipidome is an important branch of metabolome and also a distinct discipline, owing to the uniqueness and functional specificity of lipids. It involves the identification and quantification of large-scale of cellular lipid molecular species and their interactions with other metabolites in biological systems, as reviewed in [1, 2].

As an important pathway of cellular energy metabolism, lipid metabolism is correlated with the development of many diseases, including cardiovascular disease, obesity, diabetes and cancer [3–5]. The association between lipid metabolism and colorectal cancer (CRC) has been revealed during the past decades [6, 7], whereas the topic involving lipidome and cancer is emerging and has yet rarely been systematically summarized. In this review we will discuss the latest findings and emerging concepts on the impact of the changed lipidome in colorectal cancer, and suggest potential targets for clinical trials.

## LIPIDS IN CRC

**Figure 1:**
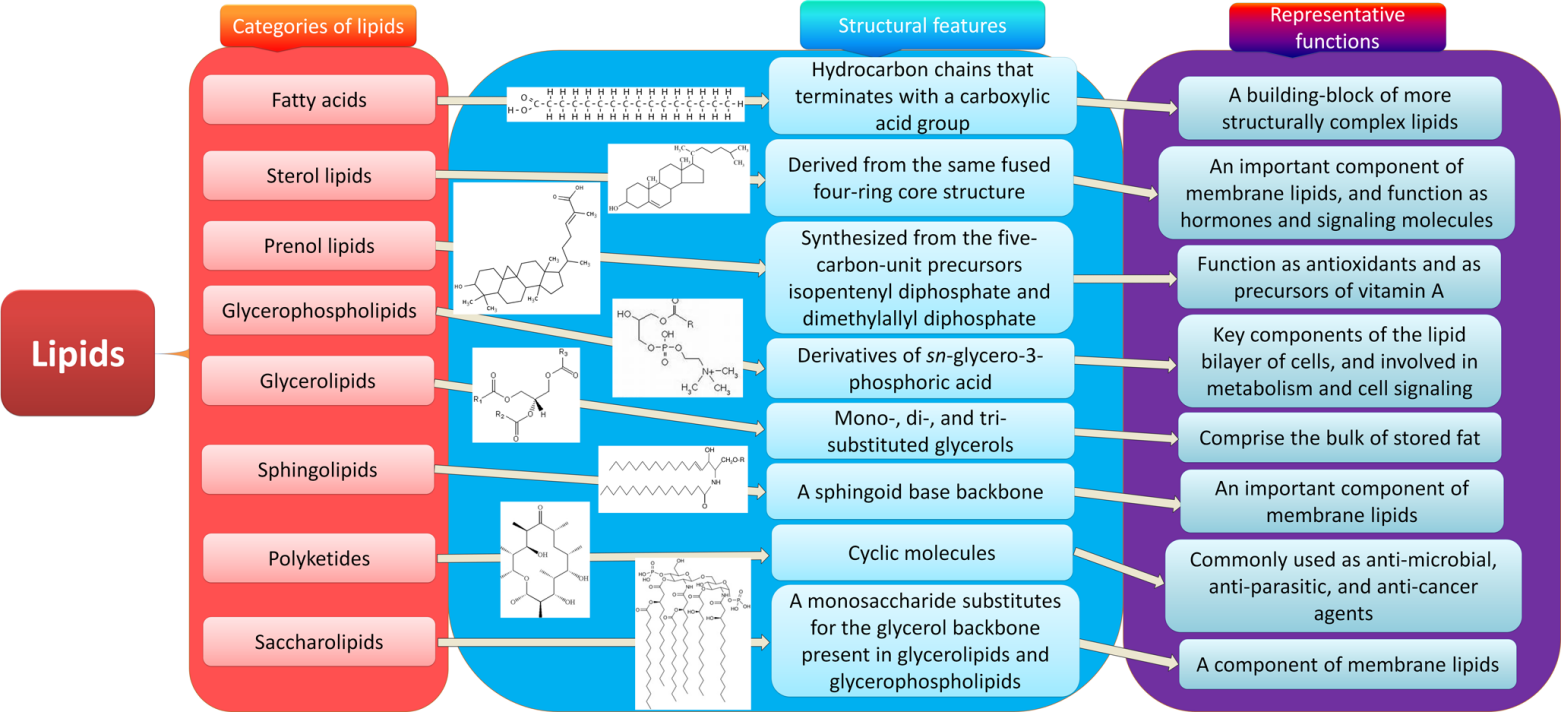
Eight categories of lipids Lipids are a group of naturally occurring molecules. There are eight categories of lipids: 1) fatty acids, a building-block of more structurally complex lipids; 2) glycerolipids, comprising the bulk of storage fat, including mono-, di-, and tri-substituted glycerols; 3) glycerophospholipids, the key components of the lipid bilayer of cells involved in metabolism and cell signaling; 4) sphingolipids, an important component of membrane lipids especially in central nervous system; 5) sterol lipids, an important component of membrane lipids, and also function as hormones and signaling molecules; 6) prenol lipids, synthesized from the five-carbon-unit precursors isopentenyldiphosphate and dimethylallyldiphosphate, function as antioxidants and as precursors of vitamin A; 7) saccharolipids, a monosaccharide substitutes for the glycerol backbone present in glycerolipids and glycerophospholipids, also a component of membrane lipids; 8) polyketides, synthesized by polymerization of acetyl and propionyl subunits, which are commonly used as anti-microbial, anti-parasitic, and anti-cancer agents.

**Figure 2:**
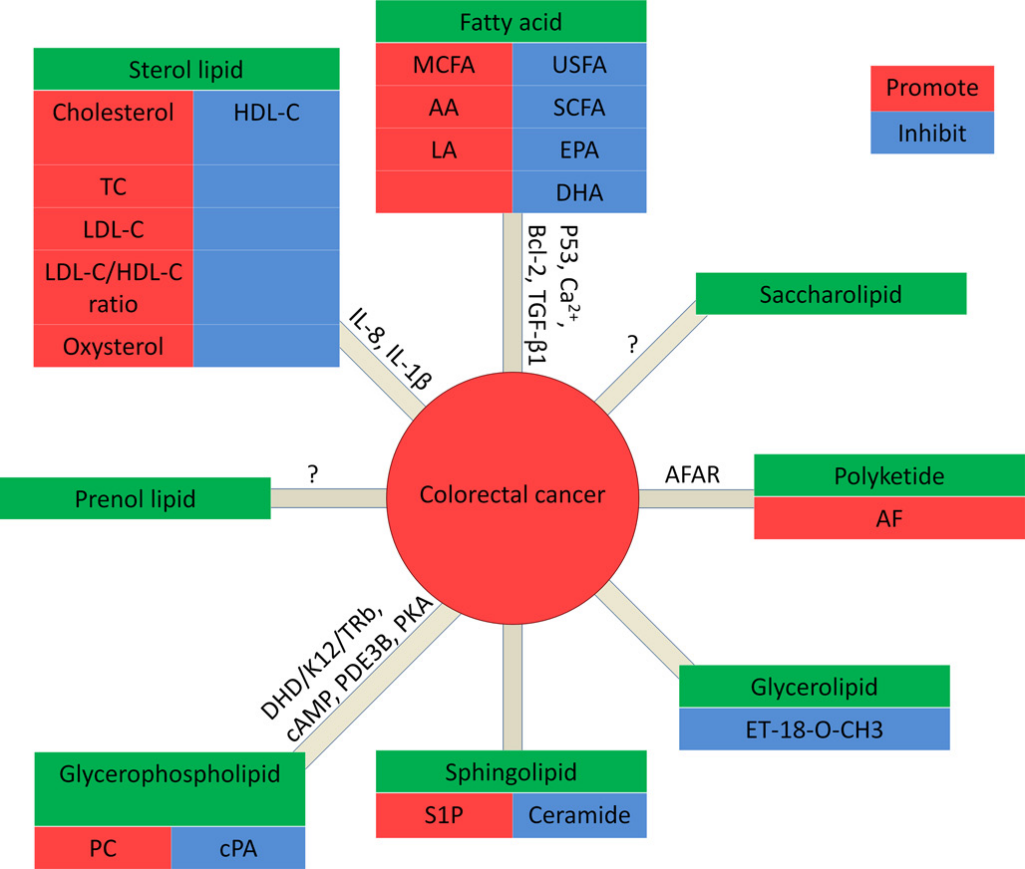
Lipids in CRC In fatty acids: MCFA, AA and LA promote CRC, while USFA (unsaturated fatty acid), SCFA (short chain fatty acid), EPA and DHA inhibit CRC. In glycerolipids: ET-18-O-CH3 promotes CRC. In glycerophospholipids: PC promotes CRC, while cPA inhibits CRC. In Sphingolipids: S1P promote CRC, while ceramide inhibits CRC. In sterol lipids: cholesterol, TC, LDL-C, and the ratio of LDL-C/HDL-C are positively correlated to CRC, while HDL-C inhibits CRC. Prenol lipids and saccharolipids: no data is available for the correlation with CRC. In polyketides: AF promotes CRC.

Lipids are a group of natural compounds comprising fats and lipoids that are insoluble in water and soluble in organic solvents. According to the structure characteristics, they are divided into eight categories: fatty acids, sterol lipids, prenol lipids, glycerophospholipids, glycerolipids, sphingolipids, polyketides and saccharolipids (Figure 1). Lipids not only serve as a source of energy, structural components of various cell membranes, but also play important roles in cytokine biosynthesis, cell signaling, energy metabolism, material transportation, cell proliferation, differentiation and development [8, 9]. The roles of lipids from the eight categories in CRC are discussed respectively below (Figure 2).

### Fatty acids

Fatty acids (FAs) are composed of long hydrocarbon chains capped by carboxyl groups (COOH). FAs differ in many flavors, based on the length of the chain and the number of double bonds. Intake of saturated FAs (SFAs) promotes cardiovascular diseases (CVD) and cancer, whereas unsaturated FAs (UFAs) may have health benefits [10–12]. Total plasma concentrations of saturated, monounsaturated and essential FAs and their polyunsaturated derivatives in CRC patients were found to be significantly lower than those in healthy controls [13]. Consistently, consumption of a high-fat diet (HFD, high in SFAs) or a low-fat diet (LFD, low in SFAs) differentially affects the development and prognosis of CRC. HFD induces oxidative stress, inflammation and cell proliferation that contribute to cancer [14, 15]. By altering the methylation patterns on insulin metabolism-related genes, HFD feeding initiates excessive insulin production and cancerous polyps [16]. In contrast, LFD reduces adipose tissue mass and inhibits proliferation of tumor cells, thereby decreasing the morbidity and risk of recurrence in cancer patients [17]. However, a LFD intervention could not reduce the risk of CRC and invasive breast cancer in postmenopausal women, during 8.1 years of follow-up [18, 19].

Dietary ω3 (*e.g*., EPA and DHA) and ω6 (*e.g*., AA) polyunsaturated FAs (PUFAs) are two major families of essential fatty acids. The former are generally associated with protective effects against colon cancer, while the latter have adverse effects [20–23]. The ω3 PUFAs can reduce cancer cell growth and differentiation by suppressing AA and the downstream eicosanoid biosynthesis [24, 25]. They also inhibit colon carcinogenesis by decreasing cyclooxygenase-2 (COX-2) and p21^ras^ expression, whereas ω6 PUFAs have the opposing effect [26, 27]. Interestingly, some ω6 PUFAs, *e.g.*, dihomo-γ-linolenic acid (DGLA), γ-linolenic acid (GLA), and linoleic acid (LA) have anti-cancer effects [28]. The combination of LA and butyrate induces the expression of the apoptosis inhibitor Bcl-2 in colonocytes, leading to decreased apoptosis of CRC cells [29]. On the other hand, combined administration of DHA and butyrate significantly enhances the apoptosis of human HCT116 colon cancer cells via an oxidation sensitive and mitochondrial Ca^2+^-dependent pathway [30].

In addition, peroxidation of PUFAs initiates oxidative stress and inflammation, which in turn promotes CRC [31]. FAs with *trans*-double bonds are difficult to digest and can increase the amount of cholesterol in blood. High consumption of trans-FAs can also increase the risk of CRC [32].

Taken together, these findings underline the importance of FAs in the etiology and prevention of CRC.

### Sterol lipids

The steroids (cholesterol and its derivatives) are a large group of natural or synthetic fatty substances with four carbon rings. Cholesterol and triglycerides (TG) are insoluble in water and their transportation is *per se* the metabolism of lipoproteins. Chylomicrons (CM) are synthesized and secreted by normal intestinal parietal cells, while very low-density lipoprotein cholesterol (VLDL) is primarily secreted from the liver. It has not been reported the correlation of CM and CRC, but VLDL levels were found positively associated with colorectal adenoma frequency [33]. In addition, CRC patients with distant metastases show significantly higher levels of TG, low-density lipoprotein cholesterol (LDL-C), and have a higher LDL-C/HDL-C (high-density lipoprotein cholesterol C) ratio, compared with patients without metastases. A nested case-control study conducted within the European Prospective Investigation into Cancer and Nutrition (EPIC) showed that the concentrations of HDL and apoA were inversely associated with the risk for colon cancer [34], suggesting that high concentrations of serum HDL are associated with decreased risk of colon cancer.

Cholesterol is also high in HFD. Increased dietary intake of total fat, cholesterol, saturated fat, and red meat is strongly associated with CRC [35]. Statins, a class of drugs that lower cholesterol by inhibiting the rate limiting enzyme in cholesterol synthesis, 3-hydroxy-3-methylglutaryl coenzyme A (HMG-CoA) reductase, thereby exerting the antiangiogenic properties [36]. Long-chain ω3 FAs can reduce the activity of HMG-CoA reductase, but cannot decrease the concentration of cholesterol in humans [37]. The mechanisms of steroid dysregulation and their association with increased CRC risk are of importance and warrant future investigation.

### Prenol lipids

The prenol lipids are composed by five-carbon-unit precursors, dimethylallyl diphosphates and/or isopentenyl diphosphates. Dolichyl phosphate functions as a potent inducer of apoptosis in rat glioma C6 cells as well as an essential carrier lipid in the biosynthesis of N-linked glycoprotein [38]. Carotenoids are important isoprenoids that function as antioxidants and as precursors of vitamin A, while vitamin K_2_ derivatives and prenylalcohols determine the tumor-specificity and control cell death [39]. Yet there is no data available for the correlation between prenol lipids and CRC.

### Glycerophospholipids

Glycerophospholipids, also called phospholipids, are the major lipids of cell membranes and key components involved in metabolism and cell signaling. The dysregulation of phospholipids in the cell membrane correlates with altered viability, proliferation, tumor development [40, 41]. *In vitro* and *in vivo* studies suggest that cyclic phosphatidic acid (cPA), a unique bioactive phospholipid, inhibits the process of mitosis and prevents the invasion and metastasis of cancer cells [42, 43]. The activity of cyclic nucleotide phosphodiesterase 3B (PDE3B), an enzyme contributes to breaking phosphodiester bonds, is also inhibited by cPA. The increase of intracellular cAMP and reduction of PDE3B activity activate the cAMP-dependent protein kinase A (PKA) pathway, which leads to the suppression of growth, proliferation, and growth of CRC [43]. In addition, The amount of phosphatidylcholine (PC) was evidently elevated in CRC cells [41]. Also, the concentration of lyso-phosphatidylcholine (LPC), the degradation product of PC, was found decreased in cancer patients using high-resolution imaging mass spectrometry. The plasma concentration of LPC correlated with weight loss [44]. These findings suggest that phospholipid levels may be used as a potential biomarker for CRC.

Phospholipids have generated strong chemotherapeutic interest, and their anti-neoplastic activity is part of a multistep process that causes ultimate cell death [45]. However, the therapeutic potential of phospholipids is low in mono-therapy studies. The possible reason is that the cytotoxic effect of phospholipids targets membranes but not DNA. Further studies are needed to explore the advantages offered by the use of phospholipids in CRC chemotherapy.

### Glycerolipids

Glycerolipids are a type of lipids composed of mono-, di-, and tri-substituted glycerols and essential for the synthesis of membrane lipids and TG. D-gluco-, D-galacto- and D-manno-configured 2-amino-2-deoxy-glycerolipids are cytotoxic to epithelial cancer cell lines and BT-474 breast cancer stem cells. The 1-O-octadecyl 2-O-methyl-sn-glycerophosphocholine (ET-18-O-CH3) exerts a highly selective cytotoxic activity against tumor cells, and causes a differential incorporation of hexadecanol into neutral ether ester glycerolipids in 2 variant rat colon carcinoma cell lines. Although ET-18-0-CH3 cannot activate the sialyltransferases during the ganglioside biosynthesis in rat colon carcinoma cells, it modifies ceramide, glycerophospholipid and neutral glycerolipid biosynthesis [46]. Therefore, the relationship between glycerolipid and CRC is of interest and remains elucidated.

### Sphingolipids

Sphingolipids are a class of lipids sharing a sphingoid base backbone and a long-chain fatty acyl CoA. Sphingolipids are structural components of cellular membranes with many important biological functions in cell growth, differentiation, death, and migration[46].

Dietary sphingolipids display both chemopreventive and chemotherapeutic effects in colon cancer animal models. Ceramide, as an important sphingolipid specie, tends to promote vascular leak and is also considered as a second messenger to transduce biological signal to suppress tumor growth. After exposed to cell stress, ceramide induces cell cycle arrest and cell death [47], while sphingosine-1-phosphate (S1P) promotes cell survival and proliferation [48]. The combination of C6-ceramide and tamoxifen can induce poly ADP ribose polymerase (PARP) cleavage, caspase-dependent apoptosis, mitochondrial membrane permeabilization, cell cycle arrest, and aggressive apoptotic responses [49]. Sphingolipids also promote TNF-related apoptosis-inducing ligand (TRAIL)-mediated apoptosis of colon cancer cells [50]. Thus, understanding the sphingolipid biology in CRC and the mechanism of sphingolipid dysregulation *in vivo* will be another potential direction for developing efficient strategies to treat CRC.

### Polyketides

Polyketides are synthesized from acetyl and propionyl subunits. Aflatoxin B1 (AFB1) is a polyketide, which is produced by the mold *Aspergillusflavus*, and known as one of the most carcinogenic compounds to hepatocellular carcinoma (HCC) [51]. The human Aflatoxin B1 aldehyde reductase (*AFAR*) gene family also maps to cancer-related genes in colorectal tumors with a smallest region of overlapping deletion [52]. In CF1 mice and Sprague Dawley rats, aflatoxins also causes pre-neoplastic alterations in the colon [53]. However, the relationship between polyketides and CRC needs further investigation.

### Saccharolipids

Saccharolipids are compounds in which fatty acids are linked directly to a sugar backbone. This unique structure makes them compatible with membrane bilayers. Saccharolipids are important cell surface markers and antigens. Yet, the roles of saccharolipids in cancer, especially in CRC are unknown.

## LIPIDOME-RELATED ENZYMES IN CRC

Enzymes involved in lipid metabolism play pivotal roles in colorectal carcinogenesis. Increased expression and subsequent higher enzymatic activity of lipogenic enzymes such as farnesyltransferase (Ftase), farnesyldiphosphate synthase (FPPS), and fat acid synthase (FAS), have been reported in CRC, compared to normal mucosa [54, 55]. The differences in activities and serum levels of lipogenic enzymes correlate with histological grading, location and stage of tumors [55]. Furthermore, the levels of lipoprotein lipase (LPL) and FAS are reduced in adjacent adipose tissue, compared to that in paired tissue distant from CRC. As a novel FAS inhibitor, oridonin induces the apoptosis and impaired viability of CRC cells *via* suppressing FAS and sterol regulatory element binding protein-1 (SREBP1) [56], which delineated FAS inhibitors as a therapeutic target drug for CRC treatment.

COX-2, a pro-inflammatory key enzyme, is responsible for the formation of prostagnoids from AA and contributes to the progression of CRC [57]. Moreover, COX-2 is over-expressed in most CRC and inflammatory bowel disease (IBD). In animal models, COX-inhibition leads to 50% reduction in carcinomas and more than 90% reduction in adenomas. Epidemiological studies also show that patients regularly taking aspirin, a COX inhibitor, have reduced risk of CRC [58]. Mice treated with lipoxygenase (LOX) inhibitors exhibit worse intestinal function during experimental colitis, compared with control [59]. These findings suggest that lipogenic enzymes are critical for the development and progression of CRC. Understanding the roles of these enzymes in individual biochemical pathways will provide molecular basis for the development of novel therapeutic interventions.

## LIPIDOME-RELATED RECEPTORS IN CRC

The knowledge of receptors driving lipid functions is also essential for understanding lipidome in CRC. Liver X receptors (LXRs) and farnesoid X receptors (FXRs) are members of the nuclear receptor superfamily (NRS) that inhibit cholesterol absorption. The activation of LXRs inhibits the proliferation of human CRC cells and the growth of intestinal tumors in mice [60], suggesting that LXR agonists are potential agents for treating CRC. Moreover, LXRα-null and FXR-null mice with high-cholesterol intake develop massive hepatic accumulation of cholesterol, while wild-type mice are resistant to cholesterol feeding [61, 62].

Prostaglandin receptors EP1, EP2 and EP4 participate in the formation of intestinal polyps and aberrant crypt foci (ACF). In contrast, the EP3 receptor pathway can counteract the effects of EP2 and EP4 receptors. The expression of EP1 and EP2 are increased in CRC tissues, while EP3 is downregulated in colon cancer cells. The selective EP3 agonist suppresses the growth of HCA-7 colon cancer cells [63].

The overexpression of BLT1 (the receptor of leukotriene B_4_, an AA metabolite via lipoxygenase pathway) is detected in human CRC tissue, while U75302, a selective BLT1 antagonist, can increase the apoptosis and decrease the proliferation of colon cancer cells [64]. Leukotriene D_4_ (LTD_4_) stimulates the proliferation of endothelial cells through cysteinyl leukotriene receptor 1 (CysLT1R) [65], which was shown expressed highly in many cancers (e.g., prostate cancer, brain cancer, neuroblastomas, and CRC) [66]. The upregulated expression of CysLT1R was correlated with a poor prognosis in patients with CRC [67]. LTD4-induced CysLT1R signaling upregulated the expression of β-catenin and COX-2 in intestinal epithelial cells, and promoted the proliferation and migration of CRC cells [68].

Lipoxin A_4_ (LXA4) and resolvin D1 (RvD1) are both PUFAs derived fatty acids *via* lipoxygenase pathways. They possess anti-inflammatory and pro-resolving actions in colitis, *via* activating their mutual receptor ALX/formyl peptide receptor 2 (FPR2) [69, 70]. ResolvinE1 (RvE1), as an antagonist against BLT1 and an agonist of ChemR23, can induce the expression of intestinal alkaline phosphatase in CRC cells, and abrogate chemically-induced colitis [71]. Due to the intense correlation between IBD and CRC, these findings indicate that pro-resolving receptors may play a beneficial role in CRC.

The G protein-coupled receptor 120 (GPR120) is a receptor of ω3 fatty acids which suppress inflammation and cancer [72, 73]. On the other hand, GPR120 is also involved in cancer progression. The expression of GPR120 was found increased in cell lines of CRC. The activation of GPR120 signaling promotes angiogenesis of CRC cells by upregulating the expression of angiogenic factors (*e.g.*, vascular endothelial growth factor (VEGF), interlukin-8 (IL-8) and PGE2) [74].

These findings indicated that the lipidome-related receptors are involved in the pathogenesis of CRC, which suggested potential targets for treating CRC.

## OTHER SIGNALING PATHWAYS INVOLVING LIPIDOME IN CRC

In addition to the above, other signaling pathways are also involved in lipidome and CRC: (a) Leptin can influence the growth and survival of CRC stem cells and possess adhesion and invasive capacities of CRC cell lines through activation of the ERK (extracellular signal-regulated kinase) and JAK signaling pathways [75]. (b) The peroxisome proliferator-activated receptor gamma (PPARG) is a nuclear receptor which regulates lipid metabolism. PPARG expressed in CRC is independently associated with longer survival of patients [76], however, its expression levels are significantly depressed in CRC compared with non-malignant tissues [77]. (c) Plasma adiponectin is associated with reduced risk of CRC, while soluble leptin receptor (sOB-R) correlates with increased risk of CRC [78]. (d) The reactive oxygen and nitrogen species (RONS) induced lipid peroxidation leads to epsilon-base DNA lesion. The concentration of NO is increased in cancerous colon tissue. The reaction between NO and O_2_^−^ can cause the formation of peroxynitrite, which participates in initiating carcinogenesis in colon cancer [79]. (e) The activation of lipid peroxidation can induce *p53* mutation, which can lead to UC (ulcerative colitis)-associated CRC [80]. (f) Melatonin released in response to lipid infusion can decrease the inhibitory effects of the ileal brake on gastric emptying and prevent CRC, ulcerative colitis, gastric ulcers and irritable bowel syndrome [81]. (g) SREBPs are strongly involved in the transcription regulation of lipogenic genes including fatty acid synthase (FAS) in colorectal cancer [82]. Modulating these important signaling pathways may provide new clues for controlling lipid metabolism and CRC progression.

## LIPIDOME IN CLINICAL TRIALS FOR CRC

Accumulating evidence suggested abnomal lipidome in CRC. Current drugs targeting lipidome against CRC also provided promising results. Due to the anti-inflammatory properties, nonsteroidal anti-inflammatory drugs (NSAIDs) have been considered as chemopreventive and therapeutic drugs for CRC [83, 84]. For example, ibuprofen reduced the risk of CRC and cellular diamine levels via augmentation of diamine efflux [85]. NSAIDs prevented frequency of carcinogen-induced animal colonic tumors [86]. However, NSAIDs also caused serious or life threatening implications on the gastrointestinal (GI) tract and other organs. Thus, it is critical to adjust the dosage of NSAIDs or combine them with other drugs to decrease the toxic effects.

15-hydroxyprostaglandin dehydrogenase (15-PGDH) is a key enzyme for eicosanoid metabolism. A recent study showed that patients who took aspirin for colon cancer prevention were less likely to develop colon cancer with a high expression of 15-PGDH. Compared with vehicle, regular aspirin use was associated with lower risk of CRC that developed within a background of colonic mucosa with high 15-PGDH expression. This study provided clues to use aspirin as a drug targeting 15-PGDH in CRC prevention [87]. Moreover, sulindac, as a broad COX inhibitor, can prevent colon cancers in 15-PGDH knockout mice [88]. Therefore, sulindac may be used as an effective agent for prevention of colon cancer with low 15-PGDH.

Abundant reports suggested that ω3 PUFAs could increase the efficacy of chemotherapy and radiotherapy of CRC. ω3 EPA also decreased markers of CD133^+^ colon cancer stem-like cells (CSLCs) while increases the sensitivity to chemotherapy [89]. A Phase II double-blind, placebo-controlled trial of EPA-FFA in patients undergoing liver resection surgery for CRC liver metastases (CRCLM) showed that EPA-FFA therapy has antiangiogenic properties and is safe and well tolerated [90]. The Phase III clinical trial of prolonged EPA treatment in CRCLM patients is warranted. Whether these specialized pro-resolving lipid mediators (SPM, *i.e*. DHA, EPA, lipoxins and resolvins) are beneficial in preventing CRC will be of particular interest to be explored.

Polyketides and their derivatives, such as epothilones, doxorubicin and mithramycin have been used as anti-cancer agents in clinics [91]. Red wine and green tea contained beneficial polyketides, which might help to lower cholesterol levels and risk of CRC. Acetogenins are unusual series of polyketides, which was selectively cytotoxic against cancerous cells and drug-resistant cancer cells [92]. Thus polyketides display promising antitumor activity against cancer both *in vitro* and *in vivo* and provide a basis for the further development of polyketide-related therapy for patients with CRC.

## CONCLUSIONS AND PERSPECTIVES

As one of the most common malignant tumors in the world, the incidence of CRC is increasing [93, 94]. Unfortunately, the prevention, treatment and prognosis remain poor for most patients due to limited understanding of its pathophysiology. The intense work is required to explain key factors impact the progress of colorectal carcinogenesis.

**Figure 3:**
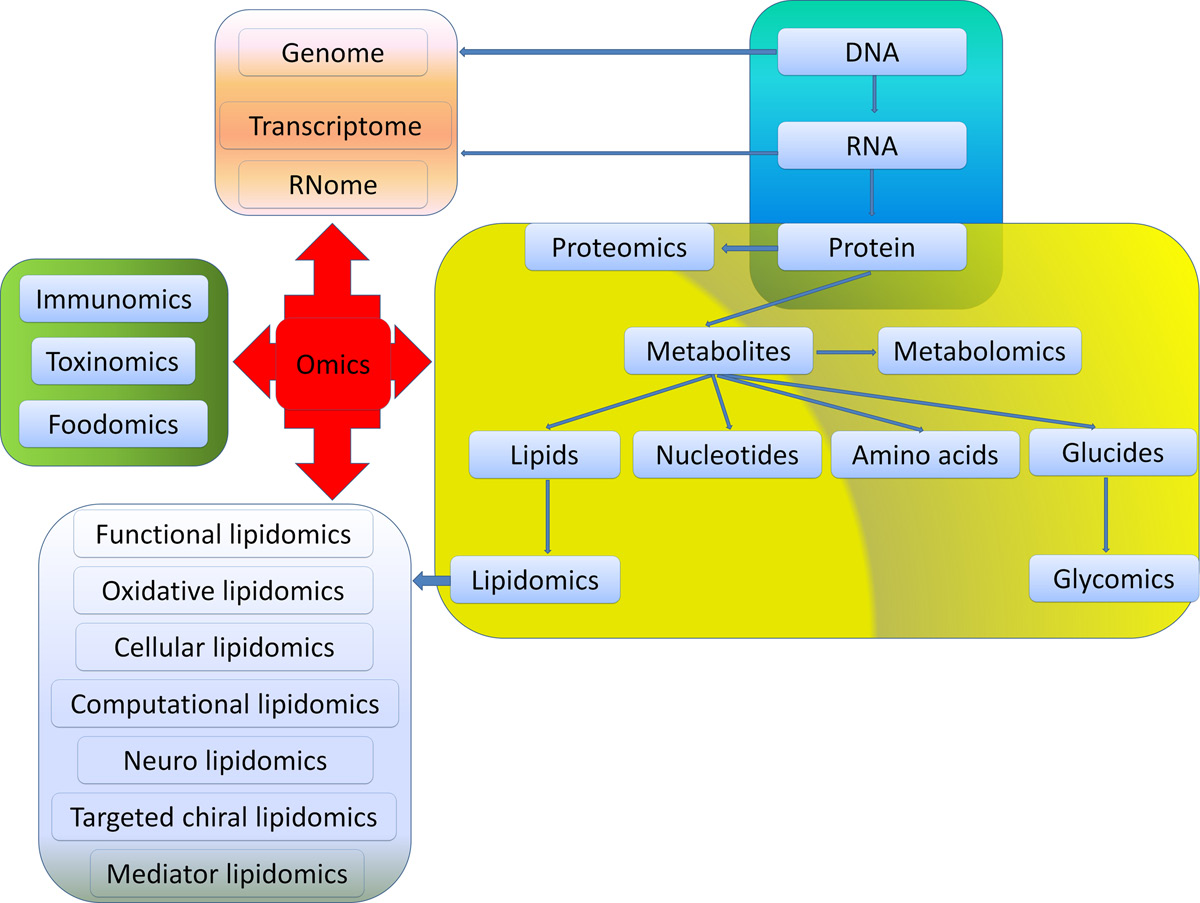
Relationships among different omics Omics is used to define a field of study in biology ending in -omics. Among them, genomics, RNomics, transcriptomics and proteomics are originated from the metabolism of DNA, RNA and protein, respectively. Metabolomics mainly focus on metabolites generated from the synthesis of DNA-RNA-Protein such as glucides, nucleotides, amino acids and lipids. Glycomics and lipidomics are included in metabolomics. Functional lipidomics, oxidative lipidomics, cellular lipidomics, computational lipidomics, neuro lipidomics, targeted chiral lipidomics as well as mediator lipidomics are derived from lipidomics. Besides, foodomics, toxinomics, and immunomics are key components in omics.

Exploring the pathogenesis of CRC and understanding the relationship between CRC and lipidome will be essential for developing new therapeutic strategies. Analyzing systemically lipidome is the most main aim of lipidomics which is an emerging discipline and methodology to research total cellular lipids and related substances in biological systems. Through comparing lipid metabolic changes in different physiological or pathological states, identifying key lipids structures, functions and interactions with other lipids, proteins and other metabolites, lipidomics reveals the mechanism of lipidome in biological metabolic regulation. However, several challenges arise. Firstly, the standard of sample preparation and extraction, the protocols of analysis, and the lipid metabolome database are not well established yet. The contaminants in samples and loss of lipids, and inconsistent approaches for analysis will inevitably lead to different results. A comprehensive database for different lipid data is essential for lipid research. This database should include encompassing structures and annotations of biologically relevant lipids, as well as lipid biology in cancers. Fortunately, the improvement of bioinformatics and biological information technology, such as Liquid chromatography tandem mass spectrometry (LC-MS/MS) and computational approaches for the prediction of lipid post translational modification, will be particularly helpful for the investigations on lipids and their impacts on the development of CRC. Secondly, understanding the complex system of lipidomics along with other omics (e.g., genomics, proteomics and metabolomics) is needed for a better comprehension of the mechanisms of colon and rectal tumorigenesis, as well as providing novel knowledge for the development of new drugs (Figure 3). Thirdly, although the biological effects of several lipids have been identified, it is still a tough and long-term task to understand the functions and metabolism of different lipids in inflammation and carcinogenesis. Fourthly, the lipidome profiling may be used as biomarkers for the diagnosis and prognosis of CRC as well as novel targets for treatment. Cancer progression involves temporal and differential regulation of multiple lipids which further amplified the complexity for investigating lipidome in CRC. Finally, the translation of lipidomics into clinical application should comply with individual treatment plans. Limited efforts have been made to use alterations in lipid metabolism to halt growth and metastases of CRC. Despite of these challenges exist, the therapies targeting lipidome will open new avenue for the diagnostics and treatment of cancer.
